# Coordinated single-cell tumor microenvironment dynamics reinforce pancreatic cancer subtype

**DOI:** 10.1038/s41467-023-40895-6

**Published:** 2023-08-26

**Authors:** Ki Oh, Yun Jae Yoo, Luke A. Torre-Healy, Manisha Rao, Danielle Fassler, Pei Wang, Michael Caponegro, Mei Gao, Joseph Kim, Aaron Sasson, Georgios Georgakis, Scott Powers, Richard A. Moffitt

**Affiliations:** 1 Department of Biomedical Informatics, Stony Brook University, Stony Brook, NY USA; 2 Department of Biomedical Engineering, Stony Brook University, Stony Brook, NY USA; 3 Department of Pathology, Stony Brook University, Stony Brook, NY USA; 4 Department of Cell Systems & Anatomy, University of Texas Health Science Center, San Antonio, TX USA; 5 Department of Pharmacology, Stony Brook University, Stony Brook, NY USA; 6 Department of Surgery, University of Kentucky and Markey Cancer Center, Lexington, KY USA; 7 Department of Surgery, Stony Brook University, Stony Brook, NY USA; 8 Stony Brook Cancer Center, Stony Brook University, Stony Brook, NY USA; 9 Department of Hematology and Medical Oncology, Emory University, Atlanta, GA USA; 10 Department of Biomedical Informatics, Emory University, Atlanta, GA USA

**Keywords:** Cellular signalling networks, Cancer microenvironment, Pancreatic cancer, Tumour heterogeneity, Tumour biomarkers

## Abstract

Bulk analyses of pancreatic ductal adenocarcinoma (PDAC) samples are complicated by the tumor microenvironment (TME), i.e. signals from fibroblasts, endocrine, exocrine, and immune cells. Despite this, we and others have established tumor and stroma subtypes with prognostic significance. However, understanding of underlying signals driving distinct immune and stromal landscapes is still incomplete. Here we integrate 92 single cell RNA-seq samples from seven independent studies to build a reproducible PDAC atlas with a focus on tumor-TME interdependence. Patients with activated stroma are synonymous with higher myofibroblastic and immunogenic fibroblasts, and furthermore show increased M2-like macrophages and regulatory T-cells. Contrastingly, patients with ‘normal’ stroma show M1-like recruitment, elevated effector and exhausted T-cells. To aid interoperability of future studies, we provide a pretrained cell type classifier and an atlas of subtype-based signaling factors that we also validate in mouse data. Ultimately, this work leverages the heterogeneity among single-cell studies to create a comprehensive view of the orchestra of signaling interactions governing PDAC.

## Abstract (web summary)

Multiple studies have characterised the tumour microenvironment (TME) of pancreatic ductal adenocarcinoma (PDAC) using single-cell RNA-seq. Here, the authors integrate the data from such single-cell studies to provide a cohesive analysis of the PDAC TME, revealing cell types and interactions that are associated with PDAC phenotypes.

## Introduction

Pancreatic ductal adenocarcinoma (PDAC) is one of the leading causes of cancer death with a 5-year survival rate of only 10%^1^. Only 1 in 4 PDAC patients are diagnosed early enough for complete surgical resection and for most patients, chemotherapy eventually becomes the only option. Molecular analysis of PDAC tumors is often hampered by limited tumor cellularity and the presence of abundant stroma intermixed with endocrine, exocrine, and immune cells^2^. To better study PDAC neoplastic cells, alternative methods such as laser capture microdissection, organoids, and xenografts have been used^3–6^. We previously performed virtual microdissection on bulk RNA-seq samples establishing prognostic gene signatures for “basal-like*”* and “classical” tumor subtypes, highlighting the importance of cancer-intrinsic heterogeneity across PDAC patients^7^. Additionally, we described “normal” and “activated” stromal subtypes, the latter having worse outcomes. Gene signatures such as these have been important in preliminary trials aimed at therapeutic decision support^8–10^. Exploration of the interplay of these signatures was recently explored and made accessible online^11^.

In recent years, the tumor microenvironment (TME) has been of great interest in the search for alternative therapeutic modalities^12^. Advances in microfluidics and single-cell RNA sequencing (scRNA-seq) have enabled high-throughput and high-resolution analysis of the cancer TME at an individual cell level^13^. This allows us to study tumor heterogeneity, while overcoming the challenges of blind-source separation^14–17^. Several single-cell studies of pancreatic tissues have helped to reveal previously unresolved biology and disease processes^18–20^. For example, novel progenitor-like duct cells were discovered that differentiate into mature ductal, acinar, or islet cells^21^. Cancer-associated fibroblasts (CAFs), including myofibroblastic CAFs (myCAFs), immunogenic CAFs (iCAFs), and antigen-presenting CAFs (apCAFs) were shown to play a role in extracellular matrix production, immunosuppression, vasculature remodeling, tumor proliferation and metastasis^22,23^. Specifically, these TME phenotypes are facilitated in part by signaling ligands) that activate or suppress transcriptional programs. Tumor-derived Interleukin-1 (IL-1), for instance, induces a cytokine cascade consisting of LIF, IL6, and G-CSF in pancreatic stellate cells (PSCs) to form iCAF populations through JAK/STAT signaling^24^. Furthermore, subgroups of macrophages and lymphocytes have been described across multiple tumor types, adding to the phenotypic diversity and transcriptional heterogeneity that ultimately allows for TME plasticity.

With many new annotated cell types, the focus has shifted towards unraveling how cell-signaling mechanisms and networks of features collectively form heterogenous tumors across PDAC patients. However, stark differences exist between cell type proportions obtained from different labs. Thus, the reproducibility of these novel cell types and unique signaling mechanisms that may exist among the tumor ecosystem and overall patient survival has not been sufficiently explored.

In this work, we leverage publicly available data sets to overcome collection and technical biases of individual analyses and create a single-cell atlas of the PDAC TME to provide a cohesive perspective of recent high-resolution findings. We demonstrate a close relationship between stromal and tumor subtypes which correlated with distinct signaling patterns across multiple cell compartments. We further highlight subtype-dependent cell-signaling interactions that may drive these distinct tissue phenotypes in hopes that targeting these interactions may lead to new therapeutic approaches.

## Results

### Integrated meta-analysis of single-cell PDAC provides a rich and detailed TME atlas

**Fig. 1:**
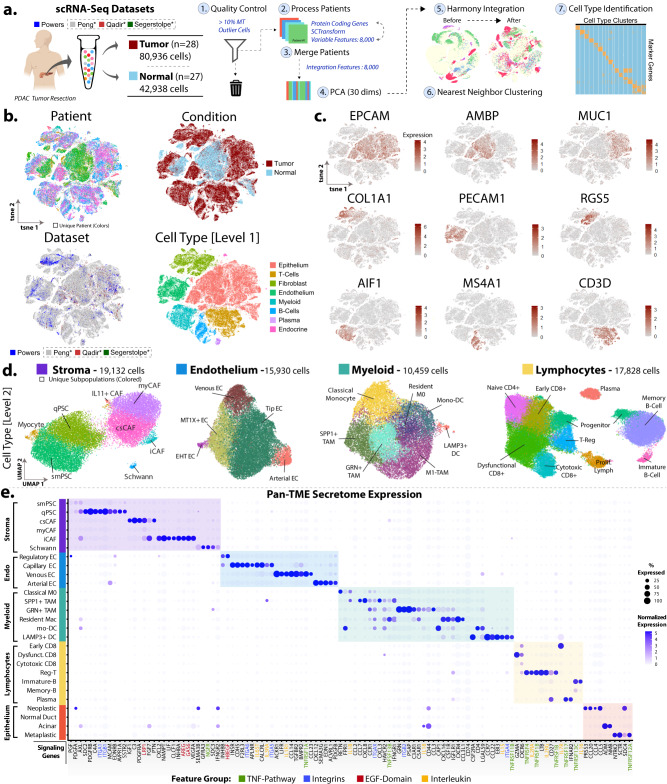
Integration of Normal and PDAC Single-Cell RNA-Seq Data. **a** Graphical workflow of the single-cell RNA-seq processing pipeline for the discovery set and atlas. A broad collection of both normal and tumor pancreas scRNA-seq data was curated by leveraging publicly available data sets. After removal of outlier cells, the standard Seurat and Harmony processing steps were performed to remove batch effects. **b** t-Distributed Stochastic Neighbor Embedding (t-SNE) projection of the full dataset showed the relative composition of the atlas based on Patients, Condition, Dataset, and Cell Type [Level 1]. **c** Canonical cell type marker genes were used to highlight specific expression of EPCAM (Epithelium), AMBP (Normal Duct), MUC1 (Aberrant Duct), COL1A1 (Fibroblasts), PECAM1 (Endothelium), RGS5 (Pancreatic Stellate Cells), AIF1 (Myeloid), MS4A1 (B-Lymphocytes), and CD3D (T-Lymphocytes). **d** Major cell type compartments (Endothelium, Stroma, Myeloid, Lymphocytes) were processed further to identify cell type [Level 2] subpopulations. **e** Differential secretome factors (ligands and receptors) expressed by each cell type subpopulation (Log-Fold Change >0.8). Expression groups are organized by the broader Cell Type 1 compartments. Color and size of dots represent % of cells expressing the gene and average expression respectively. Label colors indicate feature categories (TNF-Pathway, Integrins, EGF domain, Interleukin signaling. Source data are provided as a Source Data file.

An integrated pancreas scRNA-seq dataset of normal and PDAC-derived patients was curated using local and public data sets processed through a Seurat pipeline for both discovery and validation sets (Fig. 1a, S1a, b). Patients across data sets contributed a unique composition of cell type subpopulations reflected by the initial study objectives, patient clinical conditions, and variable tissue processing, allowing us to observe a more comprehensive set of cell types not necessarily captured by any one study (Supplementary Data 1). UMAP representation of the atlas shows the successful integration of cells across patients and independent data sets (Fig. 1b, S1c) and provides rich compositional and phenotypic information from the PDAC tissue biopsies. Visualizing canonical markers identifies the major cell types (Fig. 1c) which were further refined into distinct subpopulations for closer analysis (Fig. 1d, S1b). These cell types were additionally verified to be consistent with previously established labels from the original studies.

Fibroblastic Stroma: The desmoplastic PDAC TME is commonly represented by an increase in both activated CAFs and stellate cell populations. Analysis of differential expression (Figure S1d) between stromal subclusters identified previously described myofibroblast CAFs (myCAFs), complement-secreting CAFs (csCAFs), and immunogenic CAFs (iCAFs)^22,25^. Two other previously described pancreatic stellate cell (PSC) related subpopulations^26^ named here by smooth muscle (smPSC) and quiescent (qPSCs) were identified. Small populations of IL11+ CAFs, Schwann cells, and myocytes were observed though not present in all patients and excluded from differential gene expression analysis. Myeloid / Macrophage: We detect 2 major dendritic cell (DCs) populations and several myeloid activation states. (Figure S1e). Two dendritic populations included a Regulatory T-Cell (T-Reg) recruiting LAMP3 + DC^27^ and a monocyte-derived conventional CD1C+ DCs (mo-DCs). Resident macrophages showed complement-related expression, consistent with previous findings. Classically activated monocytes were identified by S100A8 expression^28^. We observed 3 tumor-associated macrophage (TAM) populations including a previously identified SPP1 + TAM that expressed MIF, which has been associated with alternative activation. SPP1+ TAMs expressed the highest level of CXCL8, which has been reported at the invasive tumor front^29^. An M2-like TAM subpopulation appeared to be monocyte-derived (APOC1+)^30^ and expressed granulin (GRN), which contributes to CD8+ T-cell exclusion in PDAC^31^. Endothelium: Endothelial cells were abundant within both PDAC and normal-derived pancreatic tissue. We identified 3 major cell subgroups based on previous single-cell studies, e.g., capillary ‘*Tip-like’* EC^32^, venous EC^33^, and arterial EC^34^ (Figure S1f). We identified a ‘regulatory’ endothelial subpopulation expressing anti-angiogenic markers (JAK/STAT inhibiting - SOCS3^35^, and SPRY1)^36^. Lymphocytes: We observe a dynamic landscape of both tumor-killing and tumor-permissive lymphocyte similarly observed in other solid malignancies. Collectively we identified B-lymphocytes, CD4+/CD8+ T-lymphocytes, and transitory populations such as progenitor lymphocytes and proliferating lymphocytes (Figure S1g). CD4+ T-cells included the LEF1+ Naive-CD4 and CTLA4 + T-Regs that expressed TIGIT. CD8 positivity was shared by early pre-dysfunctional TSC22D3+^37^, dysfunctional LAG3+ tumor-infiltrating lymphocytes (TILs)^38^ but decreased in the NKG7+ cytotoxic effector CD8^39^ population^40^. B-lymphocyte populations were comprised of immature (TCL1A+)^41^, mature memory B-cells, and plasma cells (MZB1+)^42^. A full list of compartment-specific gene expressions is provided in Supplementary Data 1.

### Cross-TME analysis of secretome highlights key signaling axes and surface markers

We then surveyed all differentially expressed transcripts known to be secreted ligands and receptors across the cell types described above (Fig. 1e) using statistical criteria of (Minimum cell expression > 20% of cells, expression difference between groups >10%). Notable findings include T-cell recruiting CCL21^43^ expression by qPSCs, IL6 expression by iCAFs, and INHBA by myCAFs. CXCL12, another chemotactic factor, was expressed by immunogenic CAFs and venous-ECs. The corresponding receptor, CXCR4, was highest in early (pre-activation) CD8+ T-cells and memory B-cells. The arterial, capillary, and venous endothelial subpopulations showed upregulation of angiogenic and mitogenic factors (i.e., VEGF, INSR), and immunomodulatory factors such as LIFR, CX3CL1, and CCL23. Monocyte-derived DCs expressed CSF2R, a receptor to CSF2 (GM-CSF) secreted by csCAFs and myCAFs. SPP1+ macrophages expressed MRC1 (M2 marker), and TREM2 which correlates with tumor infiltrating CD8 exhaustion^44^. LAMP3+ DCs had the highest expression of LGALS9 (Galectin-9), which promotes M2 polarization in macrophages expressing TIM-3 (HAVCR2)^45^. Lastly, we established a pan-TME panel of surface contact markers as a resource for stably expressed biomarkers that can identify cell types using orthogonal techniques (Supplementary Data 2, Figure S1i).

### Automated single-cell classifier for human and mouse data

**Fig. 2:**
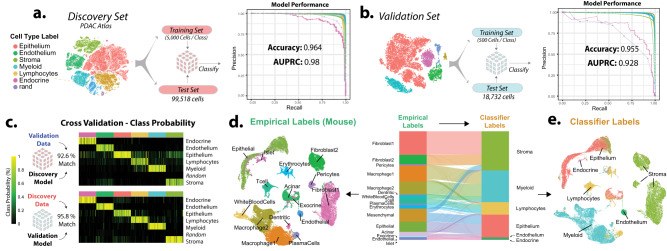
Automated single-cell classifier produces stable results in human and mouse scRNA data. **a** Training schema for the primary classifier trained on 5000 cells per cell type in the discovery set. Model performance for each cell type is shown on PR curve. **b** Secondary classifier built on an independent validation set accurately predicts the same cell type annotations. **c** Robust cross-validation of the two classifiers with unseen data are shown in the probability heatmap. **d** Sankey diagram of classifier results with mouse pancreatic cancer samples shows concordance between empirical labels and classifier labels in both human and mouse single-cell data. Width of flow is proportional to total cells in each cell category. **e** UMAP plot of mouse scRNA-seq data colored by the original independent annotations and the automated classifier results exhibit robust results. Source data are provided as a Source Data file.

To automate basic cell type annotations for pancreatic cancer single-cell data, we trained a multi-class random forest classifier using *singlecellnet*^46^ with the discovery atlas (Fig. 2a). Testing against our internal held-out validation set of 99,518 cells, we achieved model accuracy of 96.4% and AUPRC of 98%. Conversely, we trained a second random forest model using a validation dataset from three independent studies (Fig. 2b). Finally, cross-validation of both models across studies demonstrated high label correspondence (Validation: 92.6% Training: 95.8%) and probability of annotation calls within both models (Fig. 2c). To allow for compatibility with both human and mouse PDAC experimental models, we used only common features (homologous transcripts) for classification, allowing for recognition of broad cell types in both species (Fig. 2d, e).

### Normal and activated stromal signatures reflect a patient-level gradient between two distinct CAF populations

**Fig. 3:**
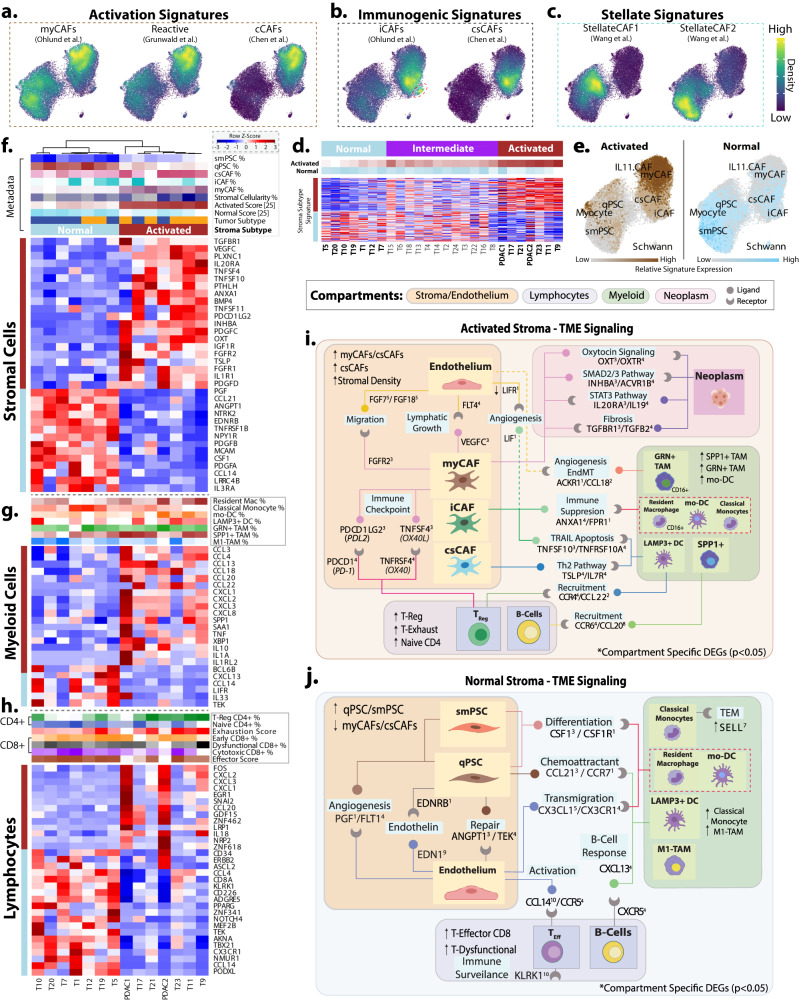
Stroma-specific variations across patients highlight distinct phenotypic groups. **a** Density enrichment of myCAFs, csCAFs, and reactive stroma signatures shows overlapping expression. **b** Enrichment of iCAF and csCAF signatures. Red circle indicates specific clustering of iCAF cells. **c** Enrichment of PSC signatures representing two separate subgroups. **d** Gene expression heatmap using Normal and Activated signatures (250 genes) clusters patients into three interpretable groups (normal, intermediate, activated) Heatmap cell colors are shown as z-score between −3 to +3. **e** Overlaying of the Moffitt Normal (blue) /Activated (brown) signature scores identified two distinct localizations of the prognostic phenotypes. **f** Pseudobulk heatmap showing differential secretome factors in the stroma between stromal subtypes. Sample tracks at the top show percentage of subpopulations in each patient stroma. **g** Heatmap showing differential secretome expression in the myeloid (monocyte/macrophages) between stromal subtypes. **h** Differential secretome/cell-contact related gene expression in the lymphocytes (T-lymphocytes) between stromal subtypes. **i** Graphical summary of the interaction network of TME signaling activities upregulated in the activated stroma. Circles and crescents represent ligands and paired receptors. Up/Down arrows indicate the compartmental subpopulation balances observed. Ligands are directional and activate the compartment and subpopulation where the corresponding receptor is distinctly expressed. **j** Graphical interaction network of upregulated signaling within the normal subtype TME. Reference to DEG: 1Fig. 1e, 2Fig S1e, 3Fig 3f, 5Fig S2g, 6Fig S1g, 7Fig S3f, 8Fig 3g, 9Fig S1f, 10Fig 3h. Heatmap colors represented by *z* score expression −3 to +3. Source data are provided as a Source Data file.

We sought to better align previously established subtypes with the more recent single-cell annotations of PDAC. We noticed a broad overlap of ‘Activated’ fibroblast signature expression involving myCAF, ‘reactive’ stroma, and classical CAFs (cCAFs)^9,22,25^ (Fig. 3a, S2a). Close examination of immunogenic subpopulations showed iCAFs and the recently described csCAFs are indeed distinct groups with overlapping signatures (Fig. 3b). Localization of the apCAF-associated CD74 expression appeared to be highest in the Schwann cells (Fig. S2b) but an isolated group of cells representing the apCAFs was not found. The remaining cells of the stroma represent 2 PSC groups with CAF1/CAF2 signatures^26^ (Fig. 3c). Furthermore, pseudobulk (aggregated) expression heatmaps of both stroma subtype signatures showed a gradient from normal stroma to gradually higher activated states. Consensus clustering divided patients into normal or activated stroma, and a mixed intermediate group (Fig. 3d, S2c, d). Stromal subtype signature scores highlighted the enrichment of the activated phenotype within myCAFs whereas the normal signature was found in PSCs (Fig. 3e).

### Cellular composition of PDAC TME is correlated with stromal subtype

Based on the distinct enrichment of subtype signatures across the CAF and PSC subpopulations, we hypothesized that differential signaling based on stromal subtypes eventually leads to distinct TME compositions and tumorigenic phenotypes (Fig. 3f–h). We observed patients of the predominantly activated stroma phenotype had with a higher percentage of myCAFs and csCAFs within the stroma while normal subtype patients had higher PSCs. Similar comparisons in the myeloid compartment showed higher SPP1+ and GRN+ TAMs, resident macrophages, and mo-DCs with activated stroma while classically activated monocytes and tumoricidal macrophages (M1-like, CIBERSORT) were higher in normal subtypes. Lymphocytes of the activated cohort showed higher proportions of CD4+ T-Regs, and higher exhausted T-cell signature scores whereas normal patients had higher overall T-cells, and an increase in CD8+ T (cytotoxic and dysfunctional), naive-CD4+ T-cells % and effector-related signature scores^47^.

### Stromal subtypes distinguish anti-tumor and pro-tumor phenotypes governed by TME signaling

In the activated subtype, myCAFs show expression consistent with secreted INHBA which is involved in the SMAD2/SMAD3 pathway in the epithelium. Additionally, the cancer progression and chemoresistance-related OXT/OXTR (oxytocin) genes were highly expressed between CAFs and the neoplastic cells^48^. Myofibroblast CAFs (myCAFs) and endothelial TGFBR1 were identified as a potential targets for TGFB2 from basal-like tumor cells^49^. IL19-induced profibrotic STAT3 pathway is shown by IL20RA in activated myCAFs^50^. Immune checkpoint molecules PDCD1LG2 (PDL2)^51^ and TNFSF4 (OX40L)^52^ were expressed by myCAFs while CD8+ T-Cells and T-Regs expressed the matching receptor pair (PD1 and OX40) respectively. Myeloid cells of the activated TME portrayed M2 polarization marked by the expression of CD209 in GRN+ TAMs (Fig. 3g). The myCAFs and endothelium, exhibited upregulation of FGF7/FGFR2 and VEGFC/FLT4 signaling that facilitates cell migration and lymphatic growth respectively^53^(Fig. 3f). The endothelium appeared to facilitate chemotaxis via IL1B signaling in the activated TME (Figure S2c) and additionally expressed macrophage regulating genes including MIF and MARCO.

Overall signaling across cells of the normal stroma cohort showed a contrasting anti-tumorigenic immune phenotype. In particular, the recruitment of cytotoxic T-Cells by the endothelium can be inferred by the upregulated CXCL1/CXCLR1 axis^54^. PSCs exhibited recruitment and differentiation through CCL21/CCR7 with LAMP3 + DC, and CSF1/CSF1R with both resident macrophages and mo-DCs. Further, we observed higher expression of NOTCH signaling and adhesion genes which may regulate TME density thereby modulating macrophage phenotypes^55^. Notably, PSCs expressed angiotensin (ANGPT1) that targets endothelial TIE2 (TEK) and facilitates vessel repair which favors the TME for drug delivery and immune infiltration^56^. The normal endothelium also showed upregulation of myeloid recruiting Fractalkine (CX3CL1)^54^. Endothelial LIFR expression was high in this cohort but down-regulated by LIF by iCAFs in the activated TME^57^ (Figure S2g). We compared the average expression of our subtype-associated panel of signaling genes across all patients (including intermediate), showing a coarse gradient of expression across subtypes (Figure S2h). Furthermore, the expression of this gene set was concordantly expressed by the stroma and myeloid compartments of the validation cohort (Figure S2i). An overall model for these interactions, based on empirical expression data, is shown in Fig. 3i, j.

### Tumor subtype signatures segregate PDAC single cells and correlate with distinct TME activity

**Fig. 4:**
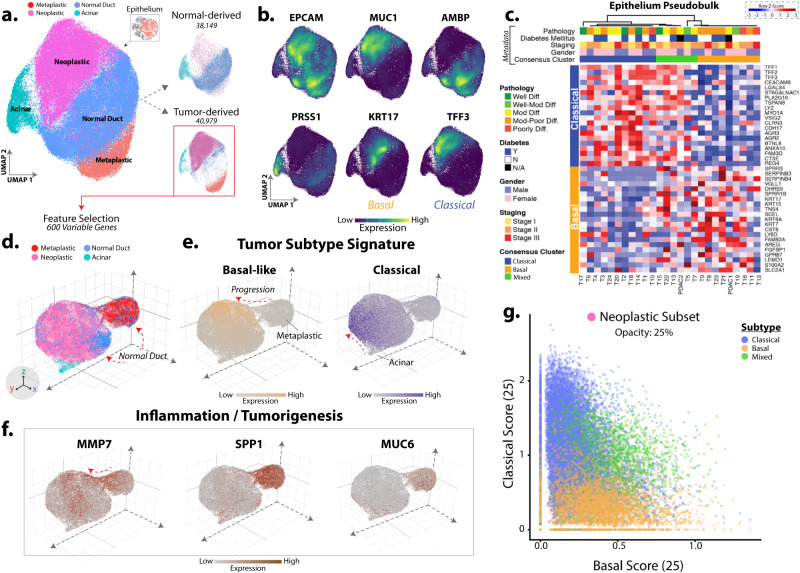
Primary PDAC subtypes show distinct localization within the epithelia. **a** UMAP projection of the integrated epithelium of both normal and PDAC-derived samples (left). Splitting this subset by source shows distinct subpopulation grouping (right). **b** Density plots show general localization of common PDAC-related markers (EPCAM: Epithelium, MUC1: Malignancy, AMBP: Normal-like, PRSS1: Acinar, KRT17: Basal-like, TFF3: Classical). **c** Expression heatmap shows the average epithelial expression (pseudobulk) for each patient (column) using the top 25 genes for both basal and classical signatures (rows). Column tracks show the associated clinical metadata and consensus clustering subtype results (blue: classical, orange: basal, green: mixed). **d** UMAP of PDAC-derived epithelia projected onto a 3D space models the separation and gradation of the transforming cells. **e** Localization of the basal-like and classical signatures scores highlight distinct regions and juxtaposition with normal duct and acinar cells. **f** Differential genes (MMP7, SPP1, MUC6) present as markers for inflammation and early precursor cells in the metaplastic subpopulation. **g** Scatterplot of the subtype scores for each single-cell in the neoplastic subset. Cells are colored by the originating patient’s subtype designation which shows distinct basal and classical groups with an additional density in between (opacity 25%). Source data are provided as a Source Data file.

Epithelial cells aggregated from both normal-derived and PDAC-derived tissue were subclustered for finer cell type annotation, revealing acinar, normal duct, inflammatory metaplastic, and neoplastic groups. Splitting the data by sample origin (normal or tumor) showed enrichment of neoplasia and metaplasia in tumor samples (Fig. 4a). UMAP density plots show clear localization of canonical genes that correspond with our cell annotations (Fig. 4b). Notably, the expression of *KRT17* (basal) and *TFF3* (classical) markers were strictly isolated to the neoplastic cluster. Epithelial analysis using pseudobulk identified patients of basal or classical phenotypes and in some cases, both were detected within the same patient’s tissue confirming recent PDAC organoids work studies of subtype dichotomy^58^ (Fig. 4c). We further analyzed the tumor epithelia using the top 1,000 variable genes and projected the data onto a 3D UMAP space (Fig. 4d). This perspective shows bridging between metaplasia and neoplasia, possibly highlighting late tumorigenic differentiation. Normal duct cells were also observed at the edge of the metaplastic cluster hinting at fundamental transitions from the major normal duct cluster (Figure S3a). The metaplastic cluster presented with early malignancy markers such as MMP7^59,60^ and MUC6^61^ (Figure S3c) and uniquely expressed tumor suppressor genes that point to a homeostatic mechanism against cellular damage or mutation (Fig. 4f). Next, we subset the neoplastic subpopulation and scored each single-cell for basal/classical subtype expression to show that cancer cells match the patient phenotype accordingly while some patients have an admixture of both basal and classical as others have observed (Fig. 4g). Cells of the ‘mixed’ subtype patients represented an intermediate state rather than merely an admixture of both neoplastic subtypes. This observation of both “hybrid” tumor patients and “intermediate” subtypes is consistent with a recent histological study^62^.

### Differential autocrine signal analysis highlights drivers of subtype-dependent TME phenotypes

Recent analysis of metastatic PDAC revealed how subtype-dependent autocrine factors can influence the TME phenotype^49^. Applying this concept to primary PDAC tissue, we present a list of differentially expressed secreted factors between basal and classical neoplastic cells (Figure S3d). We detected known basal-specific factors such as SPP1^63^ and other secreted factors that may orchestrate broader subtype-dependent changes across the ecosystem. To decrease the contribution of intermediate patient signals, the 6 intermediate patients were not included in this comparison. Particularly, factors related to epithelial-mesenchymal transition, TNFA, and IL6/JAK/STAT signaling were elevated in basal cancer cells along with signatures for inflammatory response and IFNG response.

Given the overlap of tumor and stromal phenotypes in our analysis, we wanted to outline the potential mechanisms by which tumor subtypes influence stromal phenotypes (Figure S3e). Classical cancer patients showed elevation of pancreatic intraepithelial neoplasm (PanIN) related hedgehog signaling^61^ genes (SHH, IHH) in their stroma. Correspondingly, PTCH1 was uniquely expressed in csCAFs and myCAFs pointing to an interaction between classical tumor-related desmoplasia^64,65^. Interestingly IL7, which increases immune effector cell function is unique to these patients and may lead classical tumors to have a similar cytotoxic activity seen in the normal subtype^66^. Fibroblast growth factor receptor (FGFR2), which promotes cancer stemness^67^ was highly expressed in classical epithelium^67^.

Basal-like patients had increased myCAF and csCAFs proportions and relatedly, higher activated stroma expression while classical patients showed higher PSCs. Basal-like patient stroma showed higher expression of CCL18, a chemokine known to increase T-Regs^68^ and M2-like macrophages. This immunosuppressive pattern was observed in the activated TME and CCL18 may serve as one of many mechanisms bridging PDAC tumor subtypes with stromal subtypes. BTNL8, known to stimulate proliferation and cytokine production of naive T-cells was elevated in the stroma of classical patients and may be connected to the overall increase in CD3D + T-Cells and decreased naive T-cells in these patients. Lastly, we provide differentially expressed surface-related genes for both basal and classical cancer cells to aid future investigation (Figure S3f).

### Cross-compartment-based features correlations across patients

**Fig. 5:**
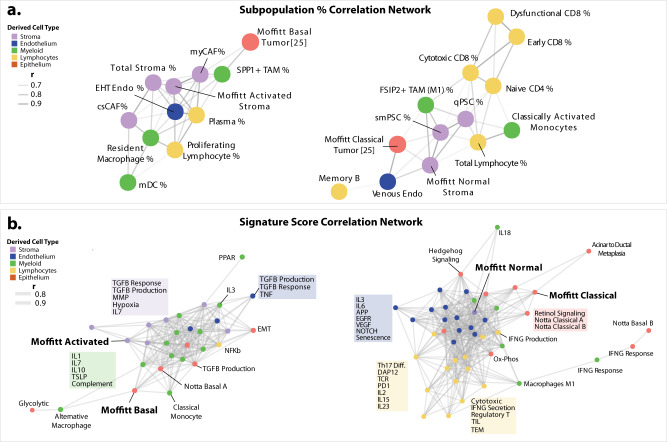
Cross-compartmental feature analysis shows two polar TME phenotype profiles. **a** Positively correlated (*r* > 0.6) feature nodes visualized on a correlation-based network layout. Node colors represent the derived Cell Type I (legend). Opacity of connections (edges) represent Pearson correlation *r* value. **b** Positively correlated (*r* > 0.7) feature nodes visualized on a correlation-based network layout. Node colors represent the derived Cell Type I (legend). Opacity of connections (edges) represents Pearson correlation *r* value). Distant nodes were adjusted to fit. The comprehensive heatmap representation of this data is presented in Supplementary Fig. 4 with significant *p* values denoted by asterisk. Source data are provided as a Source Data file.

To explore the interconnectedness of the TME, we asked how the presence of one cell type may associate with phenotypes in other compartments. Correlation analysis of subpopulation proportions revealed two major modules of correlated features (Fig. 5a, S4b). In the first module, both the normal stroma and classical cell type abundances were positively correlated. PSC stromal proportions were also significantly correlated with Classical epithelial proportions, consistent with our normal subtype observations. Notably, this group also had positive associations with cytotoxic CD8+ T-cell and the FSIP2+ TAMs. The second module grouped basal and activated signatures together along with an increase in SPP1+ TAMs, myCAFs, T-Regs, and immunogenic CAFs.

We furthermore extended this analysis with a list of gene signatures curated from recent publications (Supplementary Data 3) representing canonical PDAC subtypes, pathways, immunomodulation and metabolic signatures (Fig. 5b, S4c). When looking at compartment-specific expression signatures instead of cell type proportions, we saw ‘basal-like/activated’ signatures were associated with evidence of increased epithelial/endothelial TGFB production while the TGFB response signature was elevated in the stroma. Additionally, matrix metalloproteinase signatures within the stroma, and IL17 signaling by myeloid cells was observed (Fig. 5b). The other ‘classical/normal’ TME profile included the classical and normal subtype signatures as well as IFNG response signature elevated in the myeloid and epithelial compartment. Signatures for acinar to ductal metaplasia, hedgehog and retinol signaling were included in this module. Metabolic comparisons showed the glycolysis signature group with the basal module while oxidative phosphorylation associated with the classical module as previously described^69^. Lymphocyte signatures included transendothelial migration, tumor-infiltrating lymphocytes^70^, and PD1 signaling. Lastly, organoid-derived signatures related to positive chemotherapy response^71^ were associated with normal/classical TME. Taken as a whole, this analysis provides a uniting model of correlated cell type-specific signaling describing two drastically different TMEs in PDAC.

## Discussion

This work represents a large effort in leveraging scRNA-seq data to globally correlate published gene signatures with cell type-specific tumor composition and TME signaling. Our work indicates that previous tumor characterizations through molecular, metabolic, or histological approaches should not be viewed individually as disconnected observations. Prior work has suggested that cells of origin^72^ or lineage specifiers may control tumor subtype^73^. Additionally, recent multiplex immunofluorescence revealed striking subtype intra-tumoral heterogeneity hinting at intermediate TME landscapes^74^. Here we show co-varying modules (basal and activated vs. classical and normal) appear to orchestrate two distinct cross-compartmental paradigms, although the causal nature of this phenomenon remains unclear. While fibroblast growth factors, T-Reg dominance, and TAMs (SPP1+, GRN+) defined the activated TME, the normal TME had marked T-effector dysfunction, and endothelial transmigration and repair.

Our analysis not only corroborates established PDAC signaling phenotypes, but also highlights less studied yet interesting mechanisms that may open doors to new combination therapies. Identified as a key immunological receptor in our study, CXCR4 antagonism has been shown to allows for immune infiltration and synergistically effects with immune checkpoint inhibitors^75–77^. Inflammatory M1-like macrophages, previously shown to be dependent on NAD+ supply, was associated with the NMNAT3 + TAMs^78^. CTLA4/TIGIT+ regulatory T-lymphocytes may point to decreased effector function and increased immunosuppressive activity of lymphocytes in patients with greater activated stroma^79^. We identified IL2RG as a target enriched in classical tumor epithelium and has previously shown to attenuate PanIN growth through JAK3 suppression in orthogonally implanted pancreatic cancer^80^. Similarly, FGFR2 which promotes tumor stemness, could be targeted to block the FGF7 expressed from CAFs^81^. Moreover, the differential secretome signatures established in this work can be utilized to assess subtype-dependent secretome activity in single-cell or bulk RNA studies. By providing a pretrained cell type classifier, our work provides the building blocks of a translational platform to classify single cells, and construct TME profiles that may better inform clinicians about potential therapeutic opportunities.

Our approach to cell type labeling was conservative, aiming to avoid clusters only detectable in single studies. While we took care to avoid this pitfall, a major limitation was our reliance on batch correction of the harmony R package. With any batch correction procedures, there is a risk of introducing artifactual signals. Of note, the minor IL11+ CAFs were not identified across all studies and may be a product of the cluster-based approach in the setting of read count sparsity. A minor ‘EHT-EC’ endothelial subpopulation appeared to be low-quality cells without clear distinguishing markers. In addition, the difficulty in identifying apCAFs in our stroma meta-analysis may be related to differences in tissue pre-processing such as fibroblast disaggregation. Lastly, the validation cohort in this study was comparatively small but future studies incorporating more patients will establish the consistency of the TME patterns observed in this study.

While this manuscript derives many conclusions from expression data alone, targeted validations continue to appear across the literature. In a recent publication, Zeng et al. showed an in vitro study and immunofluorescent staining highlighting CCL18 signaling and NFKb-signaling by tumor-associated macrophages (TAMs) as an activator of stromal cells associated with poor prognosis in breast cancer patients. Appropriately, in the aggressive activated stroma concept described here, we describe a GRN + TAM-specific CCL18 signaling via ACKR1 to induce angiogenesis and endothelial-mesenchymal transition (Fig. 3j). Furthermore, our signature correlation network for the activated & basal tumor profile highlighted NFKb-signaling (specifically by lymphocytes) as a strong correlated TME feature (Fig. 5b). Additionally, Davidson et al. using CRISPR, shRNA, and flow cytometry in mouse models, showed that the immune checkpoint PD-L2 (PDCD1LG2) is predominantly found in the stroma of pancreatic cancer. Although the literature shows a heavy focus on Programmed Death Ligand expression in the context of tumor cells and lymphocytes, we found preferentially PD-L2 expression by myCAFs of the ‘activated’ TME and proposed a potential signaling axis with Regulatory T-Cells via PD1 (PDCD1) (Fig. 3i)^82^. These recent independent findings support our work, which we expect to help provide important context to future molecular studies^83–85^.

Importantly, combination therapies that target TME mediators are being investigated to expand treatment options beyond cytotoxic agents^86–88^. This is in part due to the increasing knowledge of the cancer ecosystem and improved methods for high-resolution molecular data acquisition of patient tissues and model systems. However, our ability to characterize and match patient tumors with optimal treatments hinders the development of novel therapies^89^. This study outlines a basic framework to process a single patient tumor into compartment-specific analysis that leverages pertinent gene signature related to molecular subtypes, and pathologic signaling in PDAC and other solid tumors. For example, the recent failure of a phase II clinical trial using CSF1R inhibitor, Cabiralizumab^90^, may have been related to the ‘normal stroma’ association of the CSF1/CSF1R signaling axis and non-specific targeting based on the broadly shared expression of CSF1R seen across myeloid subpopulations^91^. Future work incorporating cell-specific expression into therapeutic design may provide important context that is missing currently.

## Methods

### Ethics statement

This study was approved by Stony Brook University Human Subjects Committee (IRB), Board Ref# 2017-4223-F and 2017-4223-R1. The patients provided informed written consent to perform the comparative transcriptomic analysis.

#### PDAC tissue single-cell processing

Two PDAC patients (PDAC1 - female, PDAC2 - male) were included through approved Institutional Review Board (IRB) study #11006941 at Stony Brook Hospital. Freshly resected human PDAC tissue was acquired in the grossing room immediately following resection via Whipple or distal pancreatectomy. Bulk tissue specimen was rapidly dissociated into single-cell suspension to reduce transcriptional artifacts of handling and time. Subsequent lysis and single-cell prep were performed as detailed previously^17^. The primary tumor specimen and the metastatic lesions were delivered from the surgical pathology core, once the margins were declared negative, in advanced DMEM/F12 on ice. The specimens were washed in ice-cold PBS and minced into 5-mm^3^ sections. For each tissue section, a small section was fixed in 10% formalin for histological studies. Both the tissue specimens were processed simultaneously to avoid the batch effect. The remainder tissue specimens were minced into smaller pieces of 0.5–1 mm^3^ and digested for 45 min at 37 °C, in a solution containing 5 mg/mL Collagenase Type II (Invitrogen) and 1 mg/mL dispase (Invitrogen), in the presence of 2.5% fetal bovine serum (FBS) (Gemini Bioproducts). The tissues were additionally digested with 1 mg/mL DNAse (Stem Cell Technologies) for 15 min. The digested cells were pelleted and washed two times, and any visible blood cells were removed using the ACK lysis buffer (Sigma-Aldrich). These suspensions were filtered separately through a 70-µm cell strainer to get rid of undigested tissue chunks. If the suspension needed further declumping, it was digested for a minute in TryPLE express (Invitrogen), neutralized with 10% FBS, and filtered through a 40-µm cell strainer. The final cell suspensions were pelleted by centrifuging at 300 *g* for 5 min and resuspended in PBS with 0.1% BSA (Life Technologies) to a final concentration of 10,00,000 cells/mL. The cell viability was examined using trypan blue exclusion (Invitrogen). The Cell Ranger Single-Cell Suite 3.01 was used for demultiplexing, barcode assignment, and raw sequencing processing. The processed and unprocessed data for these patient samples is now provided as well under GEO repository (Accession #: GSE231535). Sex or gender was not used as criteria during data collection.

#### Curation of available human scRNA-seq data sets

Publicly available scRNA-seq data on pancreas was obtained from multiple sources. Raw sequencing reads from Peng et al. (GSA: CRA001160) were aligned to genes using Alevin^92^ and GRCh38 while all other data sets were integrated beginning with *gene x cell* count matrices. Additional data sets were included: Qadir et al. “GSE131886,”, Moncada et al.“GSE111672,”, Muraro et al. “GSE85241,”, Segerstolpe et al. “EMTAB-5061,”, and Lin et al. “GSE154778,”.

#### Processing and integration of human scRNA-seq Data

Seurat (v3.02 & v4.0) R package and custom scripts were used for single-cell analysis. 4 independent single-cell RNA-seq data sets were processed for quality control and preliminary analysis. Default parameters were used unless specified (min.cell = 10, min.genes = 300, percent.mt <10%). Cells with abnormally high or low feature counts, percent mitochondrial gene expression >10% were filtered. Ribosomal and mitochondrial genes were removed from features. Protein coding mRNA genes were selected as features for isolating the top 8000 highly variable genes, and the top 30 PCA dimensions were used for downstream integration. Using Harmony R package^93^, technical factors between data sets were corrected to construct a well-integrated embedding across cell types and patients. Batch-corrected harmony PCA components were then used as input for UMAP, tSNE, and FindNeighbor function. The sensitivity for FindClusters was set to 0.2 using the Louvain method. FindAllMarkers was used to annotate the coarse cell type [Level 1]. Subsequent subpopulation analysis of major cells groups (stroma, epithelium, lymphocytes, myeloid) was further dissected by applying this variable feature selection, PCA, Harmony, and dimensional reduction procedure over each cell type. Compartment-specific parameters are as follows: Myeloid (4000 variable genes, 10 dims), Epithelium (3000 genes, 8 dims), Tumor-Only subset (600 genes, 5 dims), Stroma (6000 genes, 20 dims), Endothelial (4000 genes, 12 dims), Lymphocytes (4000 genes, 18 dims). Visualization of gene expression and signature scores throughout the study used Seurat’s FeaturePlot function and nebulosa R package^94^.

#### Curation and processing of mouse scRNA-seq data sets

Curation of the mouse dataset included publicly available data sets from three different studies^23,95,96^. Mouse data sets were transformed and scaled separately before merging (i.e. Hosein, Elyada, and Gabitova-Cornell) for normalization using 3000 variable features and scaled the data sets. We selected 6,000 variable features across all data sets for integration features. 50 PCA components were used for harmony integration. UMAP parameters were as follows: assay = ”SCT”, reduction = “harmony”, dims = 1:10, n.neighbors = 80, n.component = 2, min.dist = 0.3, and negative.sample.rate = 10. Top 10 dimensions were used to find nearest neighbors and lastly 0.2 for cluster resolution. Finally, we used the FindAllMarkers function to help annotate mouse cell clusters.

#### Streamlined single-cell PDAC classifier

Using the SingleCellNet R package (v0.4.1), we trained a random forest model on the discovery atlas dataset^46^. Default parameters were used with the following exceptions (nTopGenes=20, nTopGenePairs=40, nTrees=20). For each cell type group, 5,000 *training* cells were used to establish top gene pairs. The remaining 99,518 *validation* cells were used for internal performance metrics. For cross-species utilization of the classifier, we first converted mouse gene names into human gene names trough “mouse2human” function from the R package “homologene”; if there were multiple mouse gene names aligned with one human gene name, we aggregated all into one human gene name. 14,084 common protein-coding genes within the human atlas genes and the mouse genes were set as the feature search space (original gene counts: 19,554).

#### Differential secretome analysis of TME

The atlas was down-sampled to 3000 cells for each of the subpopulations with the exception of minor cell populations that were not completely resolved (IL11+ CAFs, EHT-EC). The FindAllMarkers function was run with parameters (min.pct >0.2, min.diff.pct >0.1, method = ”MAST”). Top 100 differential genes based on log-fold change were filtered against a cell-signaling database of known secretome (685 genes), cell-contact (240 genes), ECM (85 genes) added from CellChat v0.0.2^97,98^.

#### Subtype-dependent pseudobulk analysis of TME cell signaling

Individual “pseudobulk signatures” of patients’ stroma, myeloid, endothelium, lymphocytes, and epithelial compartments were calculated by aggregating all cell expression per compartment. DESeq2 was used to define differential genes between patients with different stromal subtypes and tumor subtypes. Cell-signaling genes for subsequent translational analysis were derived once again from the CellChat v0.0.2 table of ligand/receptor interactions. Generally, candidate signaling factors activity from subpopulations was mechanistically associated with the cell type uniquely expressing the corresponding pair. Intermediate patients were excluded from both stroma and tumor comparisons. For tumor secretome analysis, intermediate patients were removed from analysis to focus on the strongest basal or classical samples. Additional code and analysis are available at https://github.com/rmoffitt/scOh.

#### Compartment phenotype correlations and networks

Cell type-specific features were calculated for all patients using an extensively curated list of gene signatures with a focus on PDAC and immune-related pathways (Supplementary Data 3). For signatures that did not have a clear cell type association, the cell type that had the highest expression across patients was selected. In a second experiment, cell type percentages were calculated for each compartment for each patient. This percentage is the fraction of a patient sample’s cell type (e.g. Stroma) that is made up of a particular cell subpopulation (e.g. myCAF). For example, the myCAF % feature represents the # of myCAFs a patient sample contains, divided by the patient’s total # of stromal cells. Further, Cytotoxic CD8+ T-Cell % is derived by a patient’s Cytotoxic CD8+ T-Cell count divided by the patient’s total # of lymphocytes. Two correlation matrices, for both signature features and cell type percentages, were produced using corrr R package. Features were sorted using ‘First Principal Component’ - sorting. Significance (designated by *) was set to *p* < 0.05. Correlation networks were produced using ggraph R package with an *r* > 0.6 and *r* > 0.7 filter for subpopulation % and signature networks, respectively. “kk” was used as the graph layout.

#### Statistics and reproducibility

The design of the study was exploratory in nature, aimed at investigating the association of tumor subtypes with individual patients’ microenvironments. No statistical method was used to predetermine sample size and data sets were integrated as they became available. To promote reproducibility and transparency in our research, we have made all data and code used for analyses publicly available.

### Reporting summary

Further information on research design is available in the Nature Portfolio Reporting Summary linked to this article.

### Supplementary information

Supplementary Information

Description of Additional Supplementary Files

Supplementary Data 1

Supplementary Data 2

Supplementary Data 3

Supplementary Data 4

Supplementary Data 5

Reporting Summary

### Source data

Source Data

## Data Availability

Transcriptomic data from newly sequenced patients are available at GEO Accession #: GSE231535. Additional publicly available data sets include Qadir et al. (GSE131886), Moncada et al. (GSE111672), Muraro et al. (GSE85241), Segerstolpe et al. (EMTAB-5061), and Lin et al. (GSE154778). Mouse data utilized in the classifier experiments include Hosen et al. (GSE12558), Elyada et al. (GSE129455), and Gabitov-Cornell et al. (GSE156210) Source data are provided in this paper.

## Code availability

Output of gene expression results, Seurat objects, patient metadata markdowns in R code, and classifier with vignette documentation can be found on GitHub: https://github.com/rmoffitt/scOh.
