# Supplementary material for: Coordinated single-cell tumor microenvironment dynamics reinforce pancreatic cancer subtype
Source: Nat Commun. 2023 Aug 26;14:5226. doi: 10.1038/s41467-023-40895-6 (PMC10460409; doi:10.1038/s41467-023-40895-6)
Supplement: Supplementary file 1 — Supplementary Information [file 41467_2023_40895_MOESM1_ESM.pdf]

Figure S1| Curation of discovery and validation datasets of PDAC sc-RNA-Seq Data

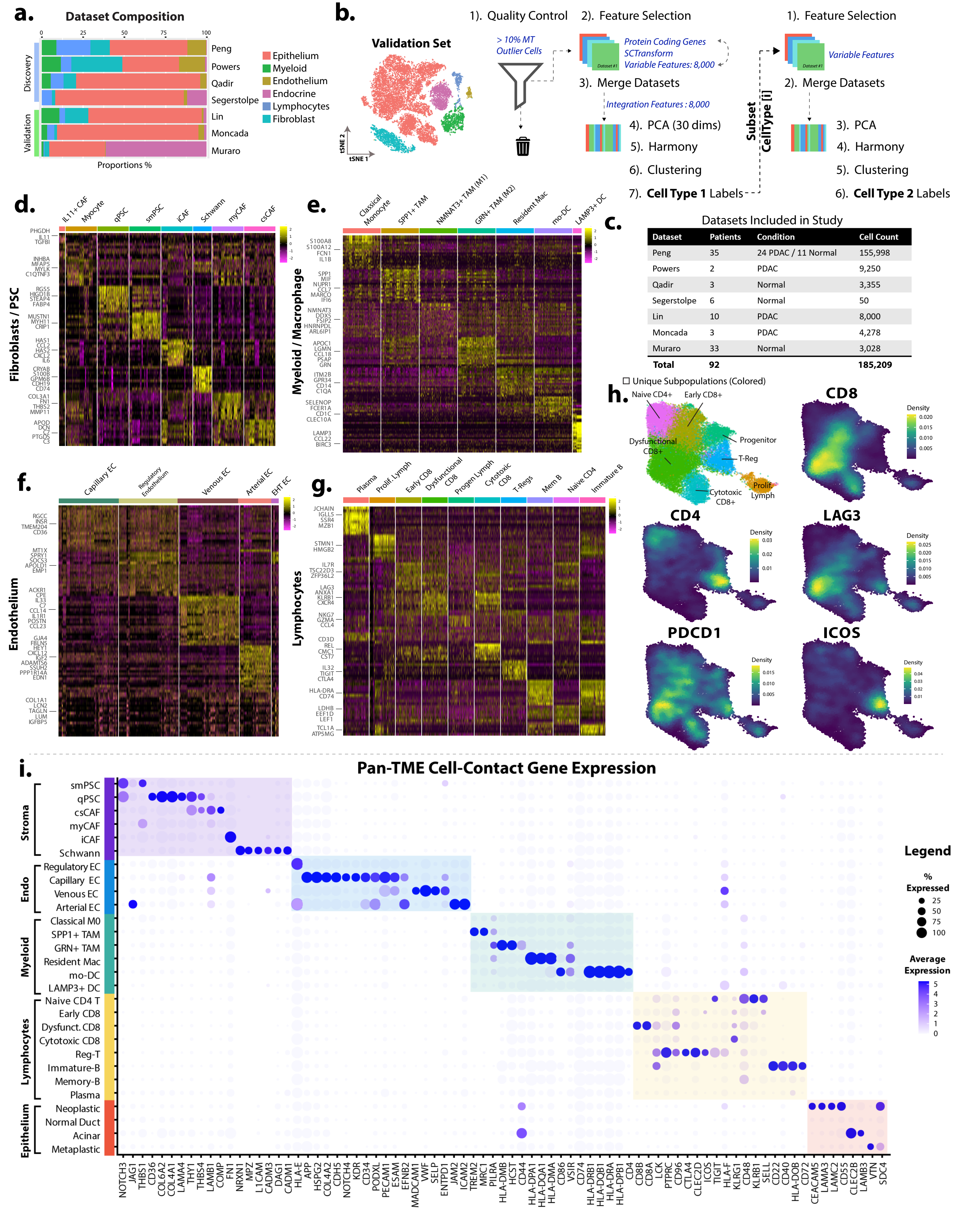

Dataset	Patients	Condition	Cell Count
Peng	35	24 PDAC / 11 Normal	155,998
Powers	2	PDAC	9,250
Qadir	3	Normal	3,355
Segerstolpe	6	Normal	50
Lin	10	PDAC	8,000
Moncada	3	PDAC	4,278
Muraro	33	Normal	3,028
**Total**	**92**		**185,209**

Figure S1 | Curation of validation set and secondary annotations a). Barplot shows the composition of major celltypes across the datasets included in the discovery and external validation set. Colored UMAP shows the cell type labels designated for each cell. **b).** Single cell data workflow outlines initial data quality controls, batch-effect removal, clustering, initial cell type annotation, and subsequent subpopulation clustering of integrated datasets. **c).** Table outlines the single cell datasets utilized in the study. **d-g).** Heatmap showing top differentially expressed (log fold change) marker genes of subpopulations within the fibroblast, myeloid, endothelial, and lymphocyte population. **h).** Further expression density plots that distinguish CD8, CD4, LAG3 (CD223), PDCD1 (PD1), and ICOS(CD278) among the lymphocytic subpopulation. (B-Lymphocytes removed for visualization). **i).** ECM and Cell-contact associated gene expression shown across the TME. (features were filtered by FC > 0.8). Source data are provided as a Source Data file.

Figure S2 | Stroma subtype associated changes across the tumor microenvironment

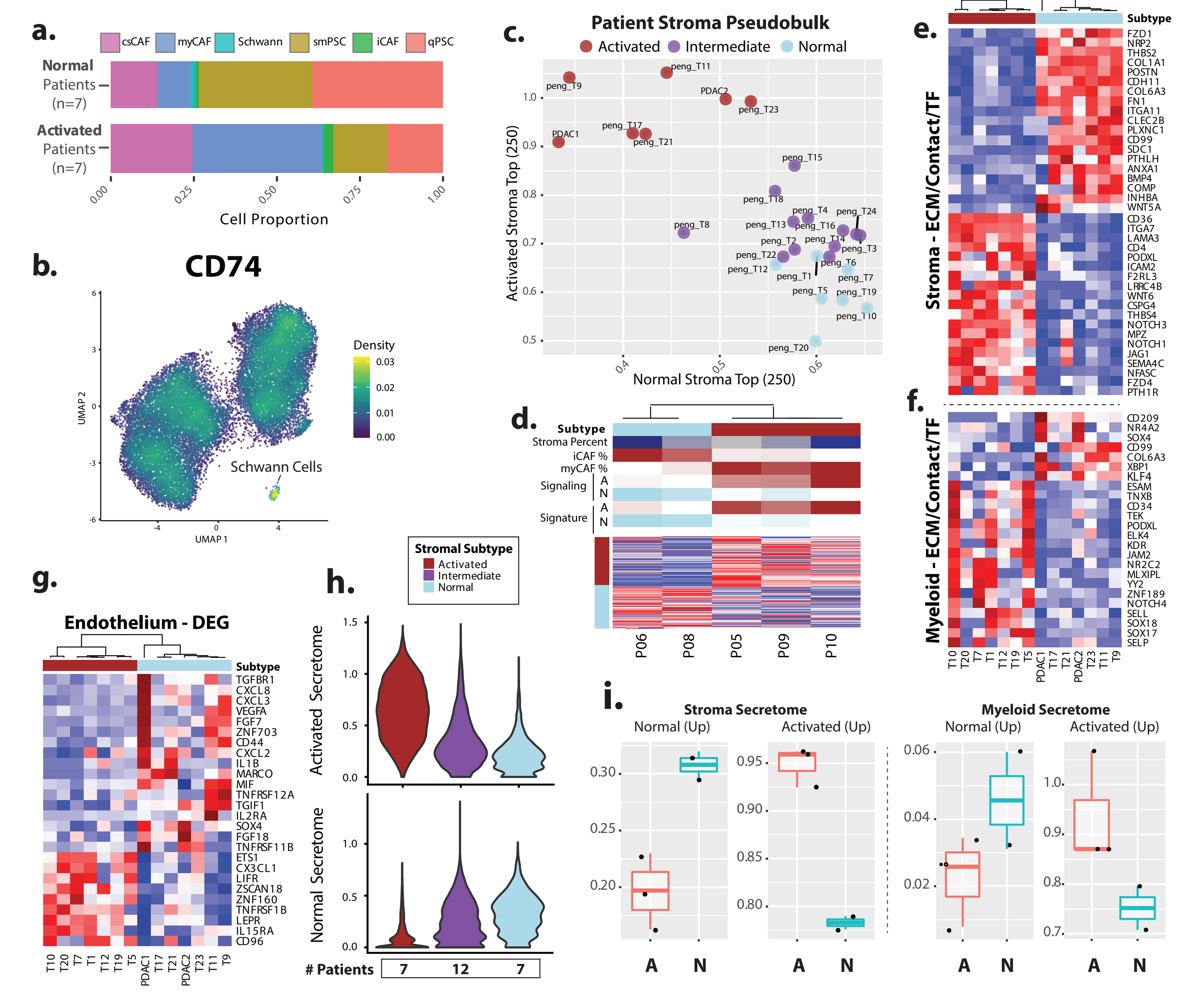

Figure S2 | Stromal subtype variations in discovery and validation set a). Segmented bar plot shows stromal cell subpopulation proportions comparison between the 7 most normal and 7 most activated patient samples. **b).** Density plot of CD74 expression in fibroblast/pericyte populations **c).** Stroma subtype signature scatterplot of discovery set samples shows purple intermediate patients which were excluded from secretome analysis. **d).** Heatmap shows subtype expression within the validation cohort. **e-f).** Heatmap showing differential ECM, cell-contact and transcription factors between the two subtypes for stroma and myeloid cells. **g).** Differential secretome heatmap of the endothelium between patient subtypes. **h).** The average expression of the top diferential secretome candidates shown across subtype groups including intermediate patients. **i).** Boxplot represents the average expression of the discovery set-based secretome candidates by the validation set; A (activated) and N (normal); Validation patient stroma (left) and myeloid compartment (right). Source data are provided as a Source Data file.

Figure S3 | Tumor subtype signatures highlights potential autocrine signaling targets

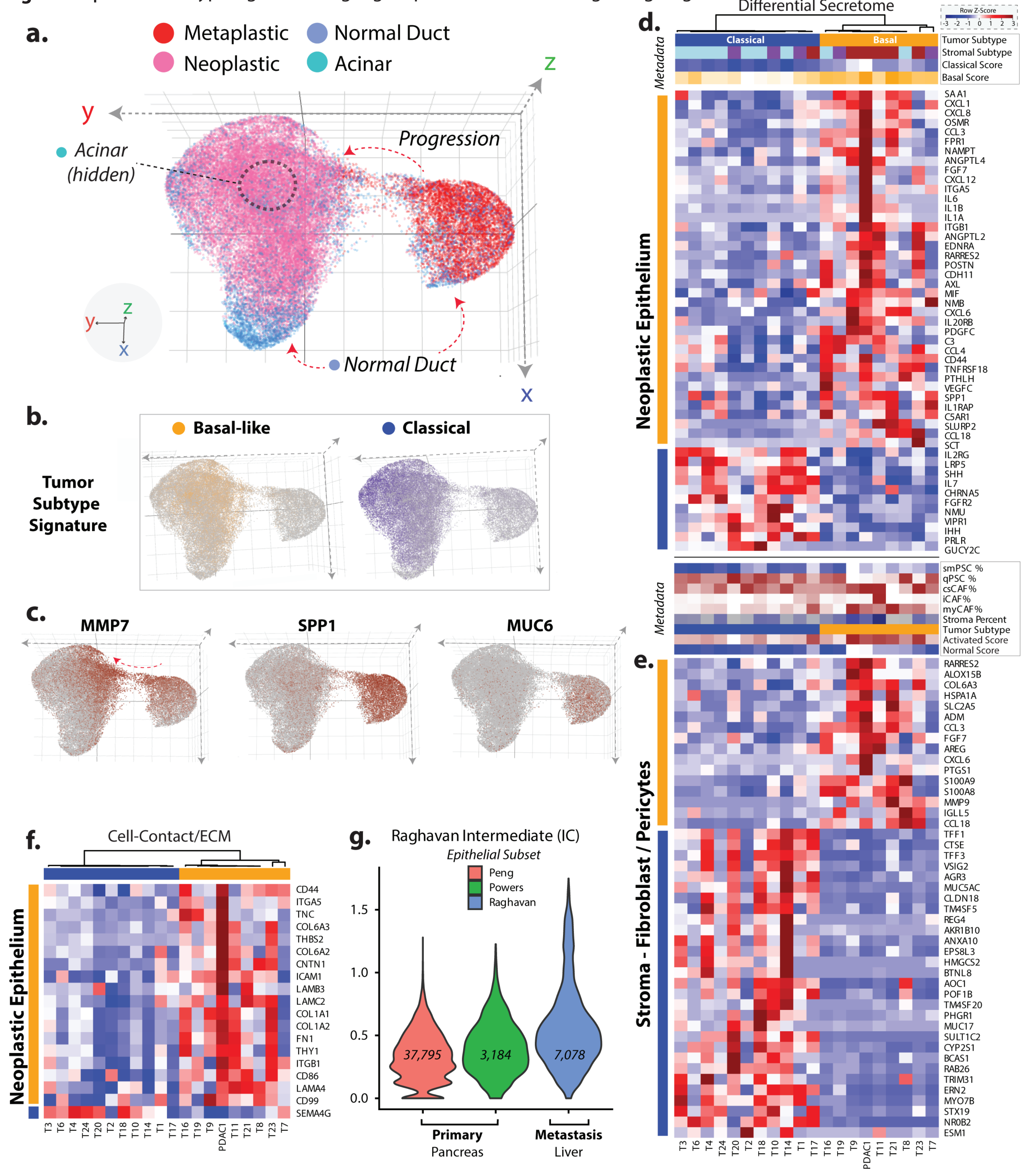

Figure S3 | Tumor subtype dependent autocrine signaling a). 3D UMAP projection of the PDAC derived epithelia with an alternate perspective highlighting potential relationships between cells. **b).** Tumor subtype signature scores (basal and classical) are enriched in spatially distinct zones. **c).** Inflammation and tumorigenesis markers are localized to the metaplastic cell cluster. MMP7 expression demonstrates a bridge between metaplasia and neoplasia (red arrow). **d).** Gene expression heatmap shows the differential autocrine signaling between the basal-like and classical cancer cells for each patient. **e).** Differential expression of patient stroma (fibroblast/pericytes) based on basal/classical subtypes. Column tracks show correlated metadata. **f).** Heatmap showing cell-contact related genes between the tumor subtypes. **g).** Violin plot shows relative signature score for intermediate (IC) between primary and metastasis derived single cell data. Source data are provided as a Source Data file.

Figure S4| Distinct TME profiles emerge based on cross compartment phenotypes

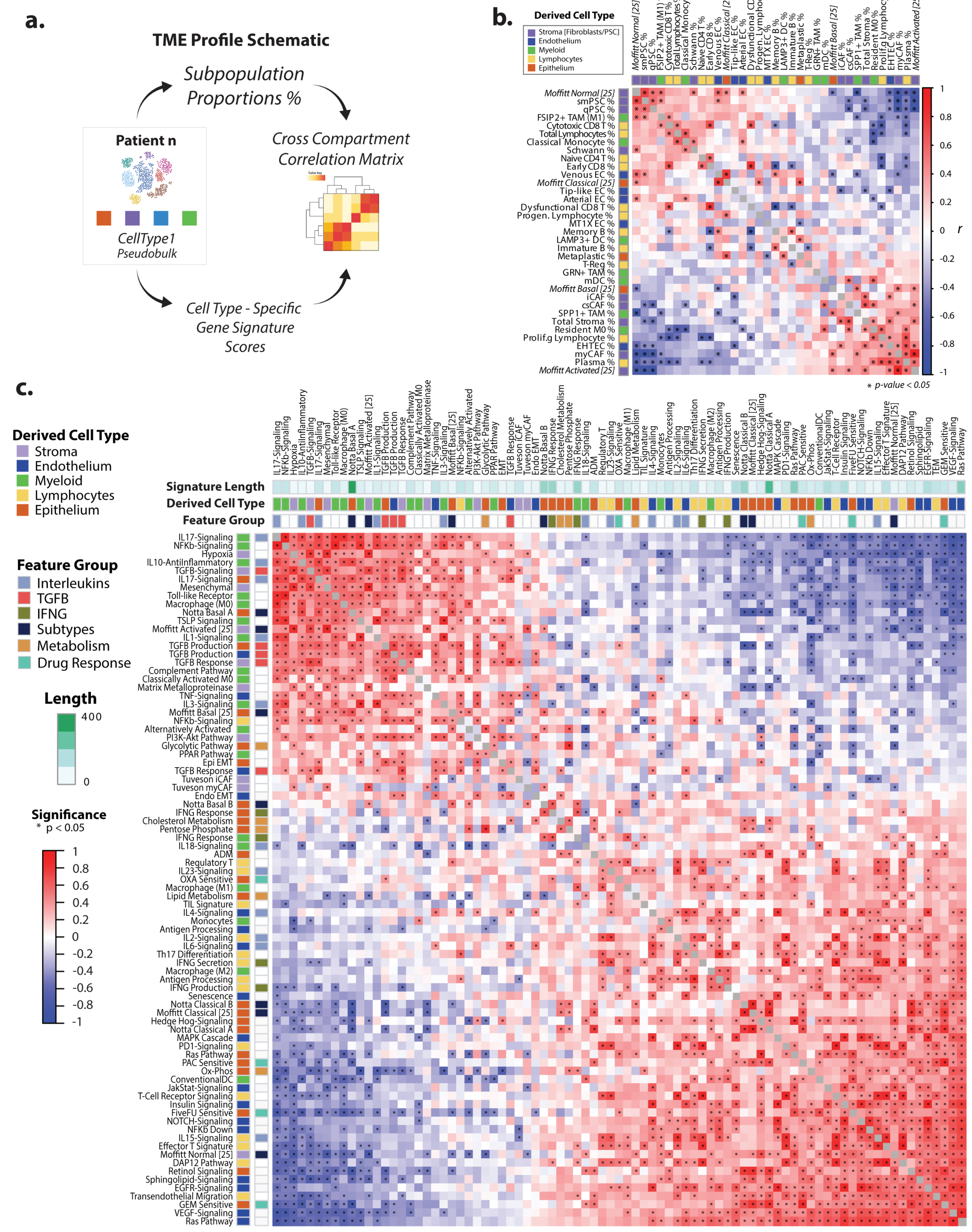

Figure S4 | Distinct TME profiles emerge based on cross compartment phenotypes a). Schematic of compartment specific feature extraction used to establish subpopulation proportions and gene signature scores for each patient. **b).** Heatmap represents the correlation matrix across subpopulation proportions. Calculated features were sorted by FPC (‘*First Principal Component*’) ordering. Colored row and column tracks represent the cell compartment used to derive % calculations. *Asterisks represent significance ($p < 0.05$). **c).** Heatmap represents the correlations of cell type specific gene signature score features measured across patients. Features were sorted by FPC (‘First Prinicpal Component’) ordering. Colored row and column tracks represent the cell compartment used to derive signature score calculations. The feature group track highlights inclusion within TME phenotypes or pathways pertinent to PDAC (*Interleukins, TGFB, IFNG, Molecular Subtype Signatures, Metabolism, Drug Response*). The ‘Signature Length’ track is shown at the top representing the # of genes found within each molecular signature. *Asterisks represent significance ($p < 0.05$). Source data are provided as a Source Data file.
