# Supplementary material for: Coordinated single-cell tumor microenvironment dynamics reinforce pancreatic cancer subtype
Source: Nat Commun. 2023 Aug 26;14:5226. doi: 10.1038/s41467-023-40895-6 (PMC10460409; doi:10.1038/s41467-023-40895-6)
Supplement: Supplementary file 2 — Description of Additional Supplementary Files [file 41467_2023_40895_MOESM2_ESM.pdf]

<u>Description of Additional Supplementary Files</u>

Supplementary Data 1 – Cell Type 2 Markers: Table of differentially expressed genes between subpopulations of cells. Differential gene outputs from Seurat objects are displayed in rows with columns representing (p-value, average log2FC, pct.1, pct.2 p_val_adjusted, cluster)

Supplementary Data 2 – Global Markers: Table of differentially expressed genes in the context of all other cell types. Differential gene outputs from Seurat objects are displayed in rows with columns representing (p-value, average log2FC, pct.1, pct.2 p_val_adjusted, cluster)

Supplementary Data 3 – Gene Lists: List of genes used/referenced in deriving signature scores. Columns show the name of gene signatures, source and genes.

Supplementary Data 4 – Dataset Metadata: Dataset overview, patient clinical metadata. Columns include Patient ID, derived condition (Normal/PDAC), Dataset origin, and Discovery/Validation allocation).

Supplementary Data 5 – Dataset Metadata Expanded: Expanded metadata showing cell populations across datasets. First tab shows number of cells split between normal and tumor derived datasets. CellType1 and CellType2 proportions are represented across datasets.
