# Supplementary material for: Coordinated single-cell tumor microenvironment dynamics reinforce pancreatic cancer subtype
Source: Nat Commun. 2023 Aug 26;14:5226. doi: 10.1038/s41467-023-40895-6 (PMC10460409; doi:10.1038/s41467-023-40895-6)
Supplement: Supplementary file 8 — Reporting Summary [file 41467_2023_40895_MOESM8_ESM.pdf]

nature portfolio

Corresponding author(s): Ki Oh, Yun Jae Yoo, Luke A Torre-Healy, Manisha Rao, Danielle Fassler, Pei Wang, Michael Caponegro, Mei Gao, Joseph Kim, Aaron Sasson, Georgios Georgakis, Scott Powers, Richard A. Moffitt*

Last updated by author(s): 2023-July 31

Reporting Summary

Nature Portfolio wishes to improve the reproducibility of the work that we publish. This form provides structure for consistency and transparency in reporting. For further information on Nature Portfolio policies, see our Editorial Policies and the Editorial Policy Checklist.

Statistics

For all statistical analyses, confirm that the following items are present in the figure legend, table legend, main text, or Methods section.

n/a	Confirmed	
☐	☒	The exact sample size (*n*) for each experimental group/condition, given as a discrete number and unit of measurement
☐	☒	A statement on whether measurements were taken from distinct samples or whether the same sample was measured repeatedly
☒	☐	The statistical test(s) used AND whether they are one- or two-sided<br>*Only common tests should be described solely by name; describe more complex techniques in the Methods section.*
☒	☐	A description of all covariates tested
☒	☐	A description of any assumptions or corrections, such as tests of normality and adjustment for multiple comparisons
☒	☐	A full description of the statistical parameters including central tendency (e.g. means) or other basic estimates (e.g. regression coefficient) AND variation (e.g. standard deviation) or associated estimates of uncertainty (e.g. confidence intervals)
☒	☐	For null hypothesis testing, the test statistic (e.g. *F*, *t*, *r*) with confidence intervals, effect sizes, degrees of freedom and *P* value noted<br>*Give P values as exact values whenever suitable.*
☒	☐	For Bayesian analysis, information on the choice of priors and Markov chain Monte Carlo settings
☒	☐	For hierarchical and complex designs, identification of the appropriate level for tests and full reporting of outcomes
☐	☒	Estimates of effect sizes (e.g. Cohen's *d*, Pearson's *r*), indicating how they were calculated

Our web collection on statistics for biologists contains articles on many of the points above.

Software and code

Policy information about availability of computer code

Data collection	External single cell data was acquired from corresponding GEO repositories. Sequence read mapping to genes for the Peng et al. dataset was performed using Alevin version 1.10.1. Internal dataset of 2 Stony Brook patients were processed using the default 10X Genomics CellRanger v 3.01 platform to quantify cell read counts.
Data analysis	Single cell data processing was performed using Seurat (v3.02 & v4.0). The Harmony R package v0.1 was used for batch correction between datasets. Subsequent downstream calculations utilized the DESEq2 R package. Custom R scripts included gene signature scoring calculations and heatmap visualizations. singleCellNet v4.1 was used for classifier training. Code and data used are available on https://github.com/rmoffitt/scOh

For manuscripts utilizing custom algorithms or software that are central to the research but not yet described in published literature, software must be made available to editors and reviewers. We strongly encourage code deposition in a community repository (e.g. GitHub). See the Nature Portfolio guidelines for submitting code & software for further information.

Data

Policy information about availability of data

All manuscripts must include a data availability statement. This statement should provide the following information, where applicable:

- Accession codes, unique identifiers, or web links for publicly available datasets
- A description of any restrictions on data availability
- For clinical datasets or third party data, please ensure that the statement adheres to our policy

Curated single cell atlas components (full, cell type specific, and down-sampled versions) have been made available on GitHub and is hosted on our publicly available server for download. Two patient samples have been deposited, now accessible through GEO Accession # GSE231535. Additional datasets were included: Qadir et al. (GSE131886) [https://www.ncbi.nlm.nih.gov/geo/query/acc.cgi?acc=GSE131886], Moncada et al. (GSE111672), [https://www.ncbi.nlm.nih.gov/geo/query/acc.cgi?acc=GSE111672], Muraro et al. (GSE85241) [https://www.ncbi.nlm.nih.gov/geo/query/acc.cgi?acc=GSE85241], Segerstolpe et al. (EMTAB-5061) [https://www.ebi.ac.uk/biostudies/arrayexpress/studies/E-MTAB-5061?accession=E-MTAB-5061], and Lin et al. (GSE154778) [https://www.ncbi.nlm.nih.gov/geo/query/acc.cgi?acc=GSE154778]. Mouse data utilized in the classifier experiments incluee Hosen et al. (GSE12558) [https://www.ncbi.nlm.nih.gov/geo/query/acc.cgi?acc=GSE125588], Elyada et al. (GSE129455) [https://www.ncbi.nlm.nih.gov/geo/query/acc.cgi?acc=GSE129455], and Gabitov-Cornell et al. (GSE156210) [https://www.ncbi.nlm.nih.gov/geo/query/acc.cgi?acc=GSE156210]. Curated single cell atlas components (full, cell type specific, and down-sampled versions) have been made available on GitHub and is hosted on our publicly

Research involving human participants, their data, or biological material

Policy information about studies with human participants or human data. See also policy information about sex, gender (identity/presentation), and sexual orientation and race, ethnicity and racism.

Reporting on sex and gender	Sex or gender was not used as criteria during data collection.
Reporting on race, ethnicity, or other socially relevant groupings	Race, ethnicity, or other socially relevant groupings were not investigated in our study.
Population characteristics	The Stony Brook Hospital research participants included two patients who had a diagnosis of pancreatic cancer (specifically Pancreatic Ductal Adenocarcinoma) and were undergoing surgical resection. The first patient was a 73 year old female with moderately differentiated Stage III PDAC. The second patient is a 60 year old male with moderately differentiated Stage III PDAC
Recruitment	Patients were asked to participate by the surgical and medical oncology team and were informed of involved pathology-based research to be performed. Participant compensation was not involved in this study.
Ethics oversight	The study was approved by Stony Brook Hospital IRB (#11006941)

Note that full information on the approval of the study protocol must also be provided in the manuscript.

Field-specific reporting

Please select the one below that is the best fit for your research. If you are not sure, read the appropriate sections before making your selection.

☒ Life sciences ☐ Behavioural & social sciences ☐ Ecological, evolutionary & environmental sciences

For a reference copy of the document with all sections, see nature.com/documents/nr-reporting-summary-flat.pdf

Life sciences study design

All studies must disclose on these points even when the disclosure is negative.

Sample size	Single cell datasets were filtered for quality. Based on unique genes expressed by each cell, the lower quartile fraction was removed. 26 total PDAC patients were initially included in subtype dependent comparisons.
Data exclusions	To derive the greatest difference between phenotypic subtypes, patients belonging to the intermediate or mixed subtypes were excluded for a portion of the study (Figure 3,4).
Replication	We designated an external validation set composed of a smaller selection of single cell datasets. We verified the ability to perform patient subtype calling, and estimation of novel secretome signatures with this test set just as we did for the primary discovery set. Cell type annotations were additionally cross-validated using this external dataset to show the consistency of our internal cell type identification process.
Randomization	*Describe how samples/organisms/participants were allocated into experimental groups. If allocation was not random, describe how covariates were controlled OR if this is not relevant to your study, explain why.*
Blinding	No blinding was involved in this study. Direct classification of each patient and the tumor/stroma subtype was required for the study.

Reporting for specific materials, systems and methods

We require information from authors about some types of materials, experimental systems and methods used in many studies. Here, indicate whether each material, system or method listed is relevant to your study. If you are not sure if a list item applies to your research, read the appropriate section before selecting a response.

Materials & experimental systems

n/a	Involved in the study
☒	☐ Antibodies
☒	☐ Eukaryotic cell lines
☒	☐ Palaeontology and archaeology
☒	☐ Animals and other organisms
☒	☐ Clinical data
☒	☐ Dual use research of concern
☒	☐ Plants

Methods

n/a	Involved in the study
☒	☐ ChIP-seq
☒	☐ Flow cytometry
☒	☐ MRI-based neuroimaging
